# Factors associated with typical enteropathogenic *Escherichia coli* infection among children <5 years old with moderate-to-severe diarrhoea in rural western Kenya, 2008–2012

**DOI:** 10.1017/S0950268820002794

**Published:** 2020-11-16

**Authors:** K. Fagerli, R. Omore, S. Kim, J. B. Ochieng, T. L. Ayers, J. Juma, T. H. Farag, D. Nasrin, S. Panchalingam, R. M. Robins-Browne, J. P. Nataro, K. L. Kotloff, M. M. Levine, J. Oundo, M. B. Parsons, K. F. Laserson, E. D. Mintz, R. F. Breiman, C. E. O'Reilly

**Affiliations:** 1 National Center for Emerging and Zoonotic Infectious Diseases, Centers for Disease Control and Prevention, Atlanta, GA, USA; 2 Kenya Medical Research Institute, Kisumu, Kenya; 3 Center for Vaccine Development and Global Health, University of Maryland School of Medicine, Baltimore, MD, USA; 4 Peter Doherty Institute for Infection and Immunity, The University of Melbourne, Melbourne, Australia; 5 Center for Global Health, Centers for Disease Control and Prevention, Nairobi, Kenya; 6 Emory Global Health Institute, Emory University, Atlanta, GA, USA

**Keywords:** Diarrhoea, *Escherichia coli* (*E. coli*), water-borne infections

## Abstract

Typical enteropathogenic *Escherichia coli* (tEPEC) infection is a major cause of diarrhoea and contributor to mortality in children <5 years old in developing countries. Data were analysed from the Global Enteric Multicenter Study examining children <5 years old seeking care for moderate-to-severe diarrhoea (MSD) in Kenya. Stool specimens were tested for enteric pathogens, including by multiplex polymerase chain reaction for gene targets of tEPEC. Demographic, clinical and anthropometric data were collected at enrolment and ~60-days later; multivariable logistic regressions were constructed. Of 1778 MSD cases enrolled from 2008 to 2012, 135 (7.6%) children tested positive for tEPEC. In a case-to-case comparison among MSD cases, tEPEC was independently associated with presentation at enrolment with a loss of skin turgor (adjusted odds ratio (aOR) 2.08, 95% confidence interval (CI) 1.37–3.17), and convulsions (aOR 2.83, 95% CI 1.12–7.14). At follow-up, infants with tEPEC compared to those without were associated with being underweight (OR 2.2, 95% CI 1.3–3.6) and wasted (OR 2.5, 95% CI 1.3–4.6). Among MSD cases, tEPEC was associated with mortality (aOR 2.85, 95% CI 1.47–5.55). This study suggests that tEPEC contributes to morbidity and mortality in children. Interventions aimed at defining and reducing the burden of tEPEC and its sequelae should be urgently investigated, prioritised and implemented.

## Introduction

Diarrhoeal diseases remain a leading cause of morbidity and mortality in young children in developing countries. In 2015, diarrhoeal diseases were estimated to have caused over 9.5 billion episodes of illness and killed nearly 500 000 children <5 years old [1].

Enteropathogenic *Escherichia coli* (EPEC) strains are diarrhoeagenic *E. coli* characterised by their ability to produce attaching and effacing lesions without carrying genes for Shiga toxins. They are capable of causing moderate-to-severe diarrhoea (MSD) in young children in developing countries [2, 3], but have rarely caused outbreaks in the USA or other industrialised nations [4]. Two sub-categories of EPEC are found in humans: typical EPEC and atypical EPEC. Typical EPEC (tEPEC) produce a type IV bundle forming pilus (*bfpA*), whereas both sub-categories include the *eae* gene [5, 6]. Previous studies have found a strong association between tEPEC and infantile diarrhoea in a number of developing countries, including Uruguay [7], Tanzania [8] and Brazil [9]. tEPEC has also been associated with excess mortality in young children [2, 10].

As with many enteric diseases, inadequate access to safe water and sanitation facilities are thought to be major contributing factors to EPEC transmission. Improved water quality, sanitation practices and hand hygiene have been found to reduce the rate of children diagnosed with EPEC infections in Brazil [11]. A 2011 report found that in Kenya, only 46% of people living in rural communities have access to improved drinking water, and only 32% of people living in Siaya County (formerly Nyanza Province) have access to improved sanitation facilities [12]. Due in large part to limited access to safe water and sanitation in this area, the reported prevalence of diarrhoea among children <5 years old was approximately 4–11% [13].

Despite the association between tEPEC and morbidity and mortality in developing countries, limited information exists on the epidemiology of tEPEC. This study aimed to describe the prevalence, clinical characteristics and sequelae of tEPEC infection, and assess associated demographic, behavioural and environmental risk factors in children <5 years old with MSD enrolled at the Global Enteric Multicenter Study (GEMS) Kenya site.

## Methods

### Study design

GEMS was a 4-year, prospective, matched case-control study that examined MSD among children <5 years old from seven sites in sub-Saharan Africa and south Asia. The GEMS Kenya site was located in Siaya County, in a portion of the Kenya Medical Research Institute's (KEMRI) and U.S. Centers for Disease Control and Prevention (CDC) Health and Demographic Surveillance System (HDSS) study area [14]. Data were collected for GEMS-1 (2008–2011) and GEMS-1A (2011–2012). The study's rationale, design, clinical and microbiologic methods, and the essential assumptions of GEMS have been previously described [15–17].

### Data collection and laboratory methods

MSD was defined as ≥3 loose stools in the previous 24 h, with onset in the previous 7 days, and ≥1 of the following characteristics: loss of skin turgor, sunken eyes, required intravenous fluid (IV) rehydration, dysentery or required hospitalisation [15].

Demographic, clinical and anthropometric data on MSD cases were collected at enrolment and at a ~60-day (range 49–90 days) follow-up home visit [18]. Data collection methods for weight and height have been further described elsewhere [16]. Height-for-age (HAZ), weight-for-age (WAZ) and weight-for-height (WHZ) *z*-scores were calculated using a WHO SAS macro and the WHO Child Growth Standards for the reference population [19, 20].

Stool specimens were taken for all children at enrolment and tested for enteric bacterial, viral and parasitic pathogens [5]. tEPEC was identified using multiplex polymerase chain reaction (PCR). For this analysis, tEPEC was defined as PCR positivity for the *eae* and *bfpA* genes. Details on the detection methods for all other enteric pathogens are described elsewhere [5].

Voluntary HIV testing, counselling and linkage to existing HIV results for mothers, fathers and case children enrolled in GEMS-1 were only available during the final 2 years of the study [16]. All children and caretakers identified as HIV positive were referred for HIV care and treatment.

### Duration of diarrhoea

At enrolment at the health facility, caretakers were asked the number of days the case-child had experienced diarrhoea on the 6 days preceding enrolment. Caretakers were also given a Memory Aid form and instructions on how to record the presence/absence of diarrhoea for the 13 days following enrolment. The total number of days of diarrhoea was calculated as the sum of these durations and distinguished by a period of 2 diarrhoea-free days, with a maximum of 20 possible diarrhoea days, which may have included multiple episodes [21].

### Verbal autopsy and health facility reported cause of death

Information on deaths was extracted from HDSS census rounds [22]. In cases where an enrolled child died at a health facility, the cause of death was ascertained from hospital records. Verbal autopsy questionnaires were carried out on all deaths of children enrolled in GEMS, whether at the health facility or in the community. For children who died at a health facility, we collected additional supplementary information based on doctors’ records.

Information from these questionnaires was entered into the InterVA version 3 or 4 program (http://www.interva.net/) and assigned a cause of death compatible with the International Classification of Diseases version 10 (ICD-10) [23].

### Statistical analysis

Data were analysed in SAS 9.4 (SAS Institute, Inc., Cary, NC). The crude associations between the sample characteristics and tEPEC were examined using *χ*^2^ or Fisher's exact test (as appropriate) for categorical variables and *t*-test for continuous variables.

To explore the factors associated with tEPEC among MSD cases, we first ran univariable logistic regressions, where each model treated tEPEC positivity as an outcome variable and each candidate factor as an independent variable. In a further step, we fit a multivariable logistic regression by including all factors with *P*-value < 0.05 from the univariable analyses to develop the final model that adjusts for potential confounding. This modelling process was repeated and performed separately for clinical characteristics, which included enrolment clinical features (32 variables), clinical characteristics at follow-up (eight variables) and environmental factors (14 variables). Age groups (0–11, 12–23 and 24–59 months) were included in all multivariable models as a categorical variable (24–59 months as a reference group); since the association with tEPEC infection and younger age has previously been reported, we report age-stratified results [2, 3].

For all anthropometric measures (HAZ, WAZ and WHZ), binary variables ( = 1, where *z*-scores were at least 2 standard deviations below the mean) were created for each as an indicator of malnutrition, and each indicator was treated as an outcome variable with tEPEC infection as an independent variable in the analysis to estimate the influence of the infection on children's growth. Outliers were excluded using WHO flags and median-absolute-deviation outlier detection methods [20, 24]. We attempted to adjust for baseline measures, but ultimately did not report these results as estimates were considered unreliable due to small sample size after excluding outliers and missing data at baseline and follow-up.

At the GEMS Kenya site, 124 case children (7.7%) were enrolled more than once, and of 135 tEPEC positive cases, 85 (63.0%) were co-infected with at least one other enteric pathogen. To examine the influence of these factors on the results, we also conducted a sensitivity analysis of models excluding repeat enrolments and subsets of multiple pathogen presence.

### Ethical review

Written informed consent was obtained in Dholuo, the predominant local dialect, from all caretakers of participating children before enrolment in the study. All study protocols, including participant HIV status collected by the CDC Division of Global HIV and TB and linked to the study, were reviewed and approved by the Scientific and Ethical Review Committees of the KEMRI (Protocol #1155) and the Institutional Review Board (IRB) at the University of Maryland, School of Medicine, Baltimore, MD (UMD Protocol #H-28327). The IRB for the CDC, Atlanta, GA deferred its review to the University of Maryland IRB (CDC Protocol #5038).

## Results

### Study enrolment and background characteristics

**Fig. 1.**
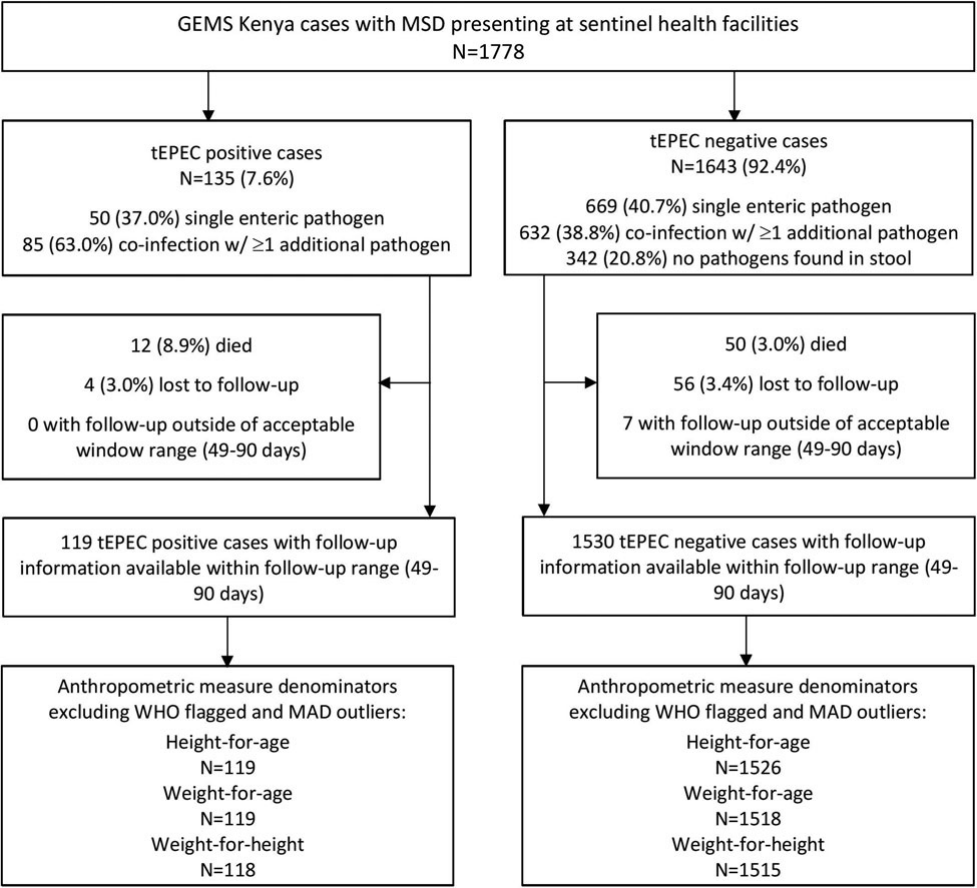
Children with MSD (*n* = 1778) by tEPEC status enrolled in GEMS, western Kenya, 2008–2012.

Of 1778 MSD cases enrolled at the GEMS Kenya site between 31 January 2008 and 30 September 2012, 135 (7.6%) stool samples were positive for tEPEC. Enteric co-infections were common, with 85 (63.0%) tEPEC cases having ≥1 additional pathogen identified in their stool (Fig. 1). Common pathogens found concurrently with tEPEC were *Giardia* (13.0%), rotavirus (12.2%) and *Cryptosporidium* (12.2%). These same three pathogens were found with similar frequencies in children without tEPEC (19%; 14% and 11%, respectively). Of the 1778 MSD cases, 1643 had stool samples negative for tEPEC and were included in the analysis.

Among all age groups, tEPEC infection rate was highest in infants (10.6% for 0–11 months, 6.1% for 12–23 months and 3.7% for 24–59 months, *P* < 0.001). Less than half of all primary caretakers completed primary school (44.8%) (Table 1). A majority of tEPEC cases lived in households that owned agricultural land and reported animals living on the compound (>90%).

**Table 1.** Demographic and household characteristics of children with MSD at enrolment, by tEPEC infection status, rural western Kenya, 2008–2012

	*N* (%)	tEPEC (+), *N* (%)	tEPEC (−), *N* (%)	*P*-value
All	1778	135 (7.6%)	1643 (92.4%)	
Age group, months				**<0.001**
0–11	829 (46.6)	88 (65.2)	741 (45.1)	
12–23	491 (27.6)	30 (22.2)	461 (28.1)	
24–59	458 (25.8)	17 (12.6)	441 (26.8)	
Gender				0.19
Male	1010 (56.8)	84 (62.2)	926 (56.4)	
Female	768 (43.2)	51 (37.9)	717 (43.6)	
Primary caretaker				0.37
Mother	1714 (96.4)	132 (97.8)	1582 (96.3)	
Other	64 (3.6)	3 (2.2)	61 (3.7)	
Primary caretaker education				0.66
Completed primary school	796 (44.8)	58 (43.0)	738 (44.9)	
Did not complete primary school	982 (55.2)	77 (57.0)	905 (55.1)	
Household characteristics				
Household size, median (range)	4 (2–17)	4 (2–12)	4 (2–17)	0.48
Number of children <5 years living in the house, median (range)	2 (1–7)	2 (1–3)	2 (1–7)	0.67
Owns agricultural land	1611 (90.6)	124 (91.9)	1487 (90.5)	0.61
Animals present in compound	1767 (99.4)	132 (97.8)	1635 (99.5)	**0.01**
Indicators of wealth				
Owns electrical assets	162 (9.1)	15 (11.1)	147 (8.9)	0.40
Has household improvements	422 (23.7)	30 (22.2)	392 (23.9)	0.67
Owns transportation	114 (6.4)	6 (4.4)	108 (6.6)	0.33

### Clinical characteristics at enrolment

Commonly reported symptoms by cases with tEPEC detected included fever (77.0%), irritable/restless (73.3%) and cough (67.4%). Twenty percent of cases with tEPEC infection received IV fluids (compared with 13.6% of MSD cases without tEPEC), and nearly that many (17.8%) were hospitalised (compared with 14.1% of MSD cases without tEPEC) (Table 2). On univariable analysis, the odds of tEPEC infection were higher for study participants with MSD who needed IV rehydration, had loss of skin turgor, abnormal hair or appeared undernourished, and among those who were offered less than normal to drink, or reportedly had convulsions, than among other study participants with MSD (all *P* < 0.05).

**Table 2.** Clinical characteristics and the OR of tEPEC infection from univariable and multivariable logistic regression models in children with MSD at enrolment, rural western Kenya, 2008–2012

	tEPEC (+), *N* (%)	tEPEC (−), *N* (%)	Univariable analysis, OR (95% CI)	*P*-value	Final model^a^, aOR (95% CI)	*P*-value
All	135	1643	–	–	–	–
Measures by clinician						
Child hospitalised	24 (17.8)	231 (14.1)	1.32 (0.83–2.10)	0.24		
IV rehydration	27 (20.0)	223 (13.6)	1.59 (1.02–2.48)	**0.04**	1.00 (0.61–1.65)	0.99
Bipedal oedema	2 (1.5)	20 (1.2)	1.22 (0.28–5.28)	0.79		
Flaky skin	6 (4.4)	43 (2.6)	1.73 (0.72–4.14)	0.22		
Loss of skin turgor	54 (40.0)	352 (21.4)	2.45 (1.70–3.52)	**<0.001**	2.08 (1.37–3.17)	**<0.001**
Sunken eyes	127 (94.1)	1538 (93.6)	1.08 (0.52–2.27)	0.83		
Under nutrition	19 (14.1)	145 (8.8)	1.69 (1.01–2.83)	**0.04**	1.06 (0.52–2.18)	0.87
Abnormal hair	12 (8.9)	73 (4.4)	2.10 (1.11–3.97)	**0.02**	1.63 (0.68–3.90)	0.28
Fever^b^	104 (77.0)	1262 (76.8)	1.01 (0.67–1.54)	0.95		
Health status^c^						
Child is thirsty	113 (83.7)	1378 (83.9)	0.99 (0.61–1.59)	0.99		
Difficulty drinking/unable to drink	8 (5.9)	56 (3.4)	1.79 (0.83–3.83)	0.14		
Offered less to drink than normal	74 (54.8)	752 (45.8)	1.44 (1.01–2.05)	**0.04**	1.14 (0.79–1.65)	0.49
Offered less to eat than normal	108 (80.0)	1320 (80.3)	0.98 (0.63–1.52)	0.92		
Difficulty breathing	30 (22.2)	310 (18.9)	1.23 (0.80–1.88)	0.34		
Cough	91 (67.4)	1000 (60.9)	1.33 (0.92–1.93)	0.13		
Belly pain	89 (65.9)	1035 (63.0)	1.14 (0.79–1.65)	0.50		
Vomit >3 times	75 (55.6)	783 (47.7)	1.37 (0.96–1.95)	0.08		
Irritable/restless	99 (73.3)	1159 (70.5)	1.15 (0.77–1.71)	0.49		
Decreased activity/lethargy	73 (54.1)	821 (50.0)	1.18 (0.83–1.67)	0.36		
Lost consciousness	15 (11.1)	133 (8.1)	1.42 (0.81–2.50)	0.22		
Rectal straining	14 (10.4)	145 (8.8)	1.20 (0.67–2.21)	0.55		
Rectal prolapse	3 (2.2)	23 (1.4)	1.60 (0.47–5.40)	0.45		
Convulsions	6 (4.4)	29 (1.8)	2.59 (1.06–6.35)	**0.04**	2.83 (1.12–7.14)	**0.03**
Condition of stool						
Contains mucus	96 (71.1)	1155 (70.3)	1.04 (0.71–1.53)	0.84		
Watery	126 (93.3)	1521 (92.6)	1.12 (0.56–2.26)	0.75		
Bloody	4 (3.0)	62 (3.8)	0.93 (0.52–1.65)	0.80		

Age groups were adjusted in the final model.

a Adjusting for the all variables significant at *P* < 0.05 from the univariate analyses.

b Temperature >38 °C measured in health facility.

c Reported by caretaker.

In the multivariable model, children presenting with loss of skin turgor, a symptom of dehydration, at enrolment (adjusted odds ratio (aOR) 2.08, 95% confidence interval (CI) 1.37–3.17), and cases reporting convulsions (aOR 2.83, 95% CI 1.12–7.14) had higher odds of tEPEC infection than their respective counterparts.

### Duration of diarrhoea

The median duration of diarrhoea was 7 days for both cases with and without tEPEC infection (interquartile range (IQR): 5–10 days and 5–9 days, respectively).

### Clinical characteristics at follow-up

None of the adverse health outcomes reported, such as having subsequent episodes of diarrhoea, vomiting, dysentery or cough between enrolment and follow-up were associated with tEPEC infection (all *P* > 0.05) (Table 3). An association between tEPEC and child mortality was shown in both univariable (OR 3.09, 95% CI 1.60–5.95) and multivariable analyses (aOR 2.85, 95% CI 1.47–5.55) (Table 3).

**Table 3.** Clinical characteristics at 49–90 day follow-up and the OR of tEPEC infection from univariable and multivariable logistic regression models in children with MSD, rural western Kenya, 2008–2012

	tEPEC (+), *N* (%)	tEPEC (−), *N* (%)	Univariable analysis, OR (95% CI)	*P*-value	Final model^a^, aOR (95% CI)	*P*-value
All	131	1580				
Mortality status						
Deceased	12 (9.2)	50 (3.2)	3.09 (1.60–5.95)	**<0.001**	2.85 (1.47–5.55)	**0.002**
Symptoms reported by caretaker between enrolment and follow-up						
Diarrhoea	89 (67.9)	1067 (67.5)	1.02 (0.69–1.49)	0.92		
Vomiting	8 (13.8)^b^	116 (15.8)^c^	0.85 (0.39–1.84)	0.67		
Dysentery	1 (0.8)	44 (2.8)	0.27 (0.04–1.96)	0.20		
Cough	18 (31.0)^b^	223 (30.3)^c^	1.03 (0.58–1.83)	0.93		

Age groups were adjusted in the final model.

a Adjusting for the all variables significant at *P* < 0.05 from the univariate analyses.

b *N* = 58.

c *N* = 735.

### Verbal autopsy and health facility reported cause of death

Of 62 case-children who died (12 tEPEC+ and 50 tEPEC−), a primary cause of death reported through verbal autopsy was available for 50 (7 tEPEC+ cases; 43 tEPEC− cases). The median number of days between enrolment and death for children with tEPEC infection was 18.5 days (IQR: 5–41.5 days), and for children without tEPEC infection was 12.5 days (IQR: 7–28 days). Malaria was the most commonly reported cause of death for children with and without tEPEC (*n* = 4 (57.1%) tEPEC+ cases; *n* = 16 (37.2%) tEPEC− cases). Diarrhoeal disease was reported as the primary cause of death for 8 (16%) children without tEPEC, and none with tEPEC.

Hospital-reported cause of death was available for 19 children. Of these, severe dehydration resulting from diarrhoeal disease was reported as the primary cause of death for one of three children with tEPEC, and for 10 of 16 children without tEPEC.

### Indicators of malnutrition

Among cases <1 year old at follow-up, being underweight (OR 2.17, 95% CI 1.31–3.59) or wasted (OR 2.45, 95% CI 1.32–4.57) were positively associated with tEPEC infection (Table 4). There was no association between stunting and infection with tEPEC.

**Table 4.** ORs of adverse anthropometric outcomes at 49-to 90-day follow-up in children with MSD by age group, western Kenya, 2008–2012

	Stunting^a^			Underweight^b^				Wasting^c^	
	tEPEC+ *n* (%)	tEPEC− *n* (%)	OR (95% CI)	tEPEC+ *n* (%)	tEPEC− *n* (%)	OR (95% CI)	tEPEC+ *n* (%)	tEPEC− *n* (%)	OR (95% CI)
0–11 months	25/77 (32.5)	181/682 (26.5)	1.33 (0.80–2.21)	27/77 (35.1)	136/682 (19.9)	**2.17 (1.31**–**3.59)**	15/76 (19.7)	62/680 (9.1)	**2.45 (1.32**–**4.57)**
12–23 months	11/27 (40.7)	175/430 (40.7)	1.00 (0.45–2.21)	7/27 (25.9)	85/426 (20.0)	1.40 (0.58–3.43)	3/27 (11.1)	34/425 (8.0)	1.44 (0.41–5.02)
24–59 months	5/15 (33.3)	169/414 (40.8)	0.73 (0.24–2.16)	1/15 (6.7)	57/410 (13.9)	0.44 (0.06–3.43)	0/15 (0)	10/410 (2.4)	–

a HAZ *z*-score at least 2 standard deviations below the mean.

b WAZ *z*-score at least 2 standard deviations below the mean.

c WHZ *z*-score at least 2 standard deviations below the mean.

### Environmental characteristics

Of 135 MSD cases with tEPEC infection, 59 (43.7%) reported using an unimproved water source and 87 (64.4%) reported treating their drinking water, 96 (73.3%) had a facility for faeces disposal and 45 (33.3%) had visible faeces in their living compound. These rates were similar to ones among cases without tEPEC (Table 5). The most common type of faeces disposal facility reported was a traditional pit toilet (*n* = 1139, 64.1%).

**Table 5.** Environmental characteristics and the ORs of tEPEC infection from univariable and multivariable logistic regression models in children with moderate to severe diarrhoea, rural western Kenya, 2008–2012

	tEPEC(+), *N* (%)	tEPEC(−), *N* (%)	Univariable analysis, OR (95% CI)	*P*-value	Final model^a^, aOR (95% CI)	*P*-value
All	135	1643	–		–	
Primary source of drinking water^b^						
Unimproved water sources	59 (43.7)	684 (41.6)	1.09 (0.76–1.55)	0.64		
Water always available from main source	129 (95.6)	1518 (92.4)	0.56 (0.24–1.31)	0.18		
Child given stored drinking water	114 (84.4)	1500 (91.3)	0.52 (0.32–0.85)	**0.009**	0.67 (0.40–1.12)	0.12
Household drinking-water treatment							
Treats drinking water	87 (64.4)	1037 (63.1)	1.06 (0.73–1.53)	0.76		
Effective treatment method^c^	81 (60.5)	987 (63.8)	1.00 (0.70–1.43)	0.99		
Observed sanitation facilities							
Has facility for faeces disposal^d^	96 (73.3)^e^	1152 (72.9)^f^	1.01 (0.68–1.52)	0.93		
Visible faeces in compound	45 (33.3)	609 (37.1)	0.85 (0.59–1.23)	0.39		
Animals present in compound	132 (97.8)	1635 (99.5)	0.22 (0.06–0.82)	**0.02**	0.25 (0.06–1.00)	0.05
Hand hygiene^g^							
Wash without soap	11 (8.2)	95 (5.8)	1.45 (0.75–2.77)	0.26		
Does not wash hands before:							
Eating	33 (24.4)	265 (16.1)	1.68 (1.11–2.55)	**0.01**	1.56 (1.02–2.37)	**0.04**
Nursing	88 (65.2)	1152 (70.1)	0.79 (0.55–1.16)	0.23		
Cooking	90 (66.7)	1093 (66.5)	1.01 (0.69–1.46)	0.97		
Does not wash hands after:							
Defaecating	20 (14.8)	383 (23.3)	0.57 (0.35–0.93)	**0.03**	0.59 (0.36–0.96)	**0.03**
Cleaning child	96 (71.1)	1221 (74.3)	0.85 (0.58–1.25)	0.41		
Handling animals	117 (86.7)	1459 (88.8)	0.82 (0.49–1.38)	0.45		

Age groups were adjusted in the final model.

a Adjusting for the all variables significant at *P* < 0.05 from the univariate analyses.

b Reported by caretaker within the 2 weeks prior to enrolment.

c Effective treatment methods include: leaving water in sun, chlorine, boiling, filtering through ceramic or other water filter.

d Observed facilities for faeces disposal include: flush toilet, VIP latrine, traditional pit latrine, pour flush toilet and VIP with water seal.

e *N* = 131.

f *N* = 1580.

g Reported by caretaker without probing from the questionnaire administrators.

tEPEC infection was positively associated with the respondent reporting not washing hands before eating (aOR 1.56, 95% CI 1.02–2.37), and negatively associated with the respondent reporting not washing hands after defaecating (aOR 0.59, 95% CI 0.36–0.96).

### Breastfeeding

Of 246 cases, <6 months old with breastfeeding status available in GEMS-1, 54 (22.0%) were reportedly exclusively breastfed. The rate of tEPEC infection in MSD cases <6 months old who were exclusively breastfed was significantly lower than in those who were not exclusively breastfed (5.6% *vs.* 14.1%, *P* < 0.001).

### HIV status

Information pertaining to participants’ HIV status was available for 1043 cases, and of these 33 (3.2%) were HIV-positive. The rates of tEPEC infection in cases who were HIV+ (9.1%) and those who were HIV− (6.5%) were not significantly different (*P* = 0.48).

For all analyses conducted, removal of repeat enrolments or subsets of multiple pathogen presence did not appreciably change the results (data not shown).

## Discussion

This study sought to identify and describe factors associated with tEPEC infection in children <5 years old with MSD at the GEMS Kenya site. Consistent with previous studies, tEPEC was commonly found among infants and its prevalence decreased with age [3, 7–9]. In the original GEMS analysis, tEPEC was strongly associated with MSD in infants (attributable fraction (AF) 5.2; 95% CI 1.8–8.5) [18], and less strongly associated with MSD in 1-year-olds (AF 3.5, 95% CI 0.3–6.7) only at the Kenya site. However, in the re-testing of a large number of random samples from all GEMS sites using qPCR methodology, tEPEC was found to be a significant attributable cause of MSD among infants and 1-year-olds in all seven GEMS sites [25]. As in previous GEMS studies, we found a strong association between tEPEC and mortality in children <5 years old among MSD cases at the Kenya study site.

There are several possible explanations for why mortality was higher in children with tEPEC. First, a previous study of genomic sequences from 70 EPEC strains from all seven GEMS sites found that certain genomic clusters were associated with confirmed virulence factors, thus lending to the weight of evidence that the observed effect is true [6]. Second, a high proportion of tEPEC cases exhibited symptoms of severe dehydration, including a loss of skin turgor, and the children often required IV rehydration. The association between dehydration and mortality is well established and likely contributed to the increased mortality among tEPEC cases. Third, infant tEPEC cases were more likely to be underweight or experience wasting at follow-up. It is plausible that malnutrition was a contributing factor for these deaths [26–28]. Previous studies suggest that children with EPEC infection subsequently may have increased risk of mortality, as EPEC infection is linked to increased malabsorption rates and failure-to-thrive [26, 29]. Our findings are further supported by a recent GEMS analysis that found an increased risk of death in children younger than 24 months with tEPEC infection among MSD cases across all GEMS sites [30].

Clinical assessments at enrolment and follow-up also showed potential presenting signs and consequences associated with tEPEC. At enrolment, a loss of skin turgor and convulsions in children with MSD were associated with tEPEC infection. Loss of skin turgor was found to be significantly associated with tEPEC after multivariable modelling, suggesting that children presenting at a health facility with tEPEC infection may be more severely dehydrated at enrolment than children with other causes of MSD, increasing their risk of mortality. tEPEC infection is known to moderate a number of cellular transport processes, including sodium glucose co-transport, a key transport system for sodium absorption in response to oral rehydration treatment [31]. To our knowledge, no previous studies have found an association between convulsions and tEPEC specifically; however, other studies have found an association between gastroenteritis and convulsions [32, 33].

Although only reported handwashing practices by caretakers were significantly associated with tEPEC infection, inadequate access to an improved water source or sanitation facility was common among virtually all enrolled MSD cases, and the importance of safe water, sanitation and hygiene (WASH) practices for decreasing the incidence of diarrhoeal illness is well established [34–36]. Although these associations were not significantly associated with tEPEC infection, it is likely that these results are reflective of the general association between enteric pathogens and poor WASH practices, rather than a lack of an association between tEPEC and WASH practices. Until the early 1960s, outbreaks of tEPEC (defined as certain classical O:H serotypes) diarrhoea were commonly reported in newborn nurseries and infant paediatric wards in hospitals in the UK, USA and other countries. Transmission from infant to infant in the nursery wards was believed to be promulgated by contaminated hands of health care providers because of inadequate washing between patient contacts. It is possible that unwashed hands are a vehicle for transmission of tEPEC to infants in Kenya [28].

The tEPEC infection rate was higher among cases with HIV than without HIV, although the difference did not reach statistical significance. A previous study in the same area of Kenya from 2011 to 2013 found that tEPEC was detected significantly more frequently in HIV-infected children who presented to two hospitals with diarrhoea than in children who were not infected with HIV and presented to those hospitals with diarrhoea [37]. The lack of a statistically significant association in this study may be a result of the small sample of HIV-infected children (3.2%) in the study population, which has active programmes for HIV care and treatment and prevention of mother to child transmission of HIV [38].

Exclusive breastfeeding among children <6 months old was not common practice at the GEMS Kenya site, but it was reported even less frequently among tEPEC+ cases (5.6%), than among tEPEC− cases (14.1%). Although not statistically significant, this finding is consistent with the suggestion that exclusive breastfeeding improves immunity in young children, consequently protecting against infections such as tEPEC [26]. Breastfed infants are less likely to consume contaminated drinking water or food, and benefit from the transfer of maternal antibodies in breast milk [39, 40].

### Limitations

Limitations of this study include: (1) the results of this study may not be generalisable to all children <5 years in Kenya, as it was conducted in a single rural site in western Kenya; (2) because the study was limited to children with MSD enrolled in GEMS, the factors associated with tEPEC we observed in children with MSD who were tEPEC+ when compared to other children with MSD who were tEPEC−, may differ from those that would be observed in a comparison with healthy controls; (3) we did not adjust for multiple testing such as Bonferroni correction in the study, as we did not posit a strong assumption of an association between tEPEC and a factor because this study was exploratory in nature; (4) some clinical symptoms (including mortality) were reported in a small number of cases, the CIs of the estimates were wide so the results are warranted for replication in a larger sample size; (5) the time to seek medical care from diarrhoeal illness could affect the young children, particularly for underweight among the infants, but we lacked such data and (6) cases with tEPEC and other pathogens detected in their stool were included in the study as there were few cases in which tEPEC was the only pathogen detected. With regards to the last limitation, when we conducted a sensitivity analysis excluding cases with co-infection, our results remained similar (data not shown). Furthermore, because we separated EPEC strains into ‘typical’ and ‘atypical’ EPEC, we conducted similar analyses on atypical EPEC. However, our study did not find atypical EPEC to be a significant contributor to morbidity or mortality among the same study population.

In addition, verbal autopsy reports may have been limited by misclassification error from caretaker responses, which could have resulted in over- or underestimates of cause-specific mortality rates [41, 42]. Although hospital reported cause of death may provide a more accurate estimate of cause-specific mortality, it was only available for the three tEPEC deaths that occurred in a health facility (33.3%), whereas two-thirds of tEPEC deaths occurred at home or elsewhere. Finally, our ability to study intermittent and longer-term outcomes of tEPEC infection was limited due to relatively short-term follow-up period from 49 to 90 days. Although there are inherent limitations in the verbal autopsy data, most deaths in GEMS could be categorised either as directly attributable to diarrhoeal disease or related to sequelae of diarrhoea, including malnutrition and increased risk of secondary bacterial infections [43].

## Conclusions

Our study found tEPEC to be a significant contributor to morbidity and mortality among young children with MSD in rural Kenya when compared with infection with other pathogens. Future studies should more closely examine the risk factors and protective factors associated with tEPEC. Interventions aimed at reducing the burden of tEPEC infection and its sequelae, including vaccines and more effective treatments, as well as promoting breastfeeding and more consistent handwashing practices, and increasing access to safe water, safely managed sanitation and hygiene infrastructure, are urgently needed.

## Data Availability

The data that support the findings of this study are available in ClinEpiDB at http://clinepidb.org.
